# In vitro biosynthesis of Ag, Au and Te-containing nanostructures by *Exiguobacterium* cell-free extracts

**DOI:** 10.1186/s12896-020-00625-y

**Published:** 2020-05-29

**Authors:** Javier Orizola, Mirtha Ríos-Silva, Claudia Muñoz-Villagrán, Esteban Vargas, Claudio Vásquez, Felipe Arenas

**Affiliations:** 1 Laboratorio Microbiología Molecular, Departamento de Biología, Facultad de Química y Biología, Universidad de Santiago de Chile, Santiago, Chile; 2 Departamento de Ciencias Nucleares, Comisión Chilena de Energía Nuclear, Santiago, Chile; 3 Center for the Development of Nanoscience and Nanotechnology, Santiago, Chile

**Keywords:** *Exiguobacterium*, Metal(loid), Reduction, Aerobiosis, Anaerobiosis, Nanostructure

## Abstract

**Background:**

The bacterial genus *Exiguobacterium* includes several species that inhabit environments with a wide range of temperature, salinity, and pH. This is why the microorganisms from this genus are known generically as polyextremophiles. Several environmental isolates have been explored and characterized for enzyme production as well as for bioremediation purposes. In this line, toxic metal(loid) reduction by these microorganisms represents an approach to decontaminate soluble metal ions via their transformation into less toxic, insoluble derivatives. Microbial-mediated metal(loid) reduction frequently results in the synthesis of nanoscale structures—nanostructures (NS) —. Thus, microorganisms could be used as an ecofriendly way to get NS.

**Results:**

We analyzed the tolerance of *Exiguobacterium acetylicum* MF03, *E. aurantiacum* MF06, and *E. profundum* MF08 to Silver (I), gold (III), and tellurium (IV) compounds. Specifically, we explored the ability of cell-free extracts from these bacteria to reduce these toxicants and synthesize NS in vitro*,* both in the presence or absence of oxygen.

All isolates exhibited higher tolerance to these toxicants in anaerobiosis. While in the absence of oxygen they showed high tellurite- and silver-reducing activity at pH 9.0, whereas AuCl_4_^−^ which was reduced at pH 7.0 in both conditions. Given these results, cell-free extracts were used to synthesize NS containing silver, gold or tellurium, characterizing their size, morphology and chemical composition. Silver and tellurium NS exhibited smaller size under anaerobiosis and their morphology was circular (silver NS), starred (tellurium NS) or amorphous (gold NS).

**Conclusions:**

This nanostructure-synthesizing ability makes these isolates interesting candidates to get NS with biotechnological potential.

## Background

The genus *Exiguobacterium* is quite diverse composed by Gram-positive, facultative anaerobic, non-sporulating, and motile rods. Bacteria from this genus have been isolated from a variety of environments including permafrost, salt lakes, deserts and even industrial wastes, suggesting a high plasticity, adaptation capacity, and tolerance to extreme environmental factors [1]. The ability to grow in such harsh conditions makes them interesting candidates to develop products with applications in biotechnology and bioremediation.

In recent years, research on *Exiguobacterium* has been of great interest mainly because it is considered as a source of enzymes that exhibit a broad range of thermal stability [1–3]. Numerous enzymes from these microorganisms exhibiting interesting activities have been reported including: i) an esterase from *E. acetylicum*, stable between pH 6.0–11.0 [4]; ii) a protease from *Exiguobacterium* sp. SKPB5, thermally stable between 4 and 60 °C at pH 4.0–9.0 [5]; iii) a dehydrogenase from *Exiguobacterium* spp. F42, stable between 4 and 45 °C [6]; iv) a β-glucosidase from *Exiguobacterium sp* GXG2 with activity ranging between 5 to 35 °C [7], among others. Regarding bioremediation applications, it has been observed that *Exiguobacterium* sp. 2Sz shows great potential to remove pesticides [8], *Exiguobacterium* sp. GS1 eliminates hexavalent chromium from water over a wide range of temperature and pH [9], while *Exiguobacterium* sp. WK6 reduces arsenate to arsenite at arsenic-polluted sites [10]. Other examples include *Exiguobacterium* sp. ZM-2, which reduces chromium (VI) to chromium (III) [11] and *E. mexicanum* that produces silver-containing nanostructures [12].

Here, our aim is to characterize *Exiguobacterium* strains able to form nanostructures (NS) when they are expose to silver, gold, or tellurium salts. These metal(loid)s have not known biological function and are considered as non-essential; on the contrary, they trigger high toxicity even at very low concentrations [13, 14]. This toxicity is exerted, at least in part, through the generation of reactive oxygen species (ROS), which is well-known to induce oxidative stress in the cell that damages important macromolecules such as proteins, DNA, and membranes [14]. Cells mutants lacking enzymes involved in ROS elimination exhibit enhanced sensitivity to tellurium and iron [15, 16]. In addition, the presence of Ag or Te salts produce, directly or indirectly, [4Fe-4S] center dismantling of certain enzymes, releasing concomitantly Fe^2+^ to cytoplasm that results in increased hydroxyl radical formation through the Fenton reaction [17, 18]. Nevertheless, some microorganisms can handle the presence of these toxics using a several resistance mechanisms or cell responses, including: i) decreased production of metal(loid)- transporters [19, 20]; ii) repair of oxidation sensitive molecules through enzymes or antioxidant production [21]; and iii) chemical modification of the metal(loid)s and their reduction to elemental state (frequently less toxic) [14, 22, 23] which usually leads to NS formation.

Microbial synthesis of NS is preferred compared to other strategies, because it is considered a safe method and friendly with the environment. However, microbial-mediated metal(loid) reduction is not fully understood [24, 25]. In fact, it has been observed that metal(loid) reduction can be a result of secondary activities of certain enzymes, as the case of the glutathione reductase in *Pseudomonas* sp. BNF22 (reduces Au^3+^ and Te^4+^) [26], catalase in *Staphylococcus epidermidis* and *E. coli* (reduces Te^4+^) [27], or the nitrate reductase in *Fusarium oxysporum* (reduces Ag^+^) [28]. Since the metal(loid) ion reduction under anaerobic conditions should not render in the formation of ROS, contrary to observed in the presence of oxygen [17, 29, 30]. In this work we examined the metal(loid) resistance, reduction and the ability to synthesize NS by specific environmental *Exiguobacterium* strains, both in the presence or absence of oxygen. We evaluated if different respirations induce bacterial responses for those toxicants which could lead to differential generation of NS.

## Results

### *Exiguobacterium* metal(loid) resistance

*E. acetylicum* MF03, *E. aurantiacum* MF06 and *E. profundum* MF08 were exposed to K_2_TeO_3_, AgNO_3_ or HAuCl_4_ under both aerobic and anaerobic conditions, to determine the minimal inhibitory concentrations (MIC) for each treatment (Table 1). Under anaerobic conditions, *E. profundum* MF08 was the most resistant strain to silver (MIC 0.25 mM). In turn, *E. acetylicum* MF03 was equally resistant to silver in both conditions, aerobic and anaerobic.

**Table 1:** MICs of Ag (I), Au (III) and Te (IV) for the indicated *Exiguobacterium* strains

	***E. acetylicum*** MF03		***E. aurantiacum*** MF06		***E. profundum*** MF08	
Metal(loid)	+ O_2_	- O_2_	+ O_2_	- O_2_	+ O_2_	- O_2_
Ag (I)	0.125 ± 0.06	0.125 ± 0.06	0.0625 ± 0.034	0.125 ± 0.06	0.125 ± 0.06	0.25 ± 0.13
Au (III)	0.25 ± 0.13	0.5 ± 0.27	0.125 ± 0.06	0.5 ± 0.27	0.25 ± 0.13	0.5 ± 0.27
Te (IV)	0.25 ± 0.13	2 ± 0.3	0.25 ± 0.13	1 ± 0.5	0.5 ± 0.27	4 ± 0.4

Concentrations are expressed in mM and the data represent the average of 6 independent trials

For gold, under anaerobic conditions either *E. acetylicum* MF03 and *E. profundum* MF08 showed resistance values that were two-fold higher than those observed in aerobiosis; whereas, gold resistance of *E. aurantiacum* MF06 was 4-fold higher in the absence of oxygen.

As for tellurium, all strains were more resistant to tellurite under anaerobic conditions: 8- fold for *E. acetylicum* MF03 and *E. profundum* MF08, and and 4-fold *E. aurantiacum* MF06. In parallel to MICs determinations, all *Exiguobacterium* strains were analyzed for their metal(loid) resistance in solid medium by determining growth inhibition zones (Figure S1). Consistent with MIC determinations, all strains -excepting *E. acetylicum* exposed to silver (Figure S1 A)- showed higher resistance to these toxicants under anaerobic conditions.

### Metal(loid)-reducing activity

**Fig. 1:**
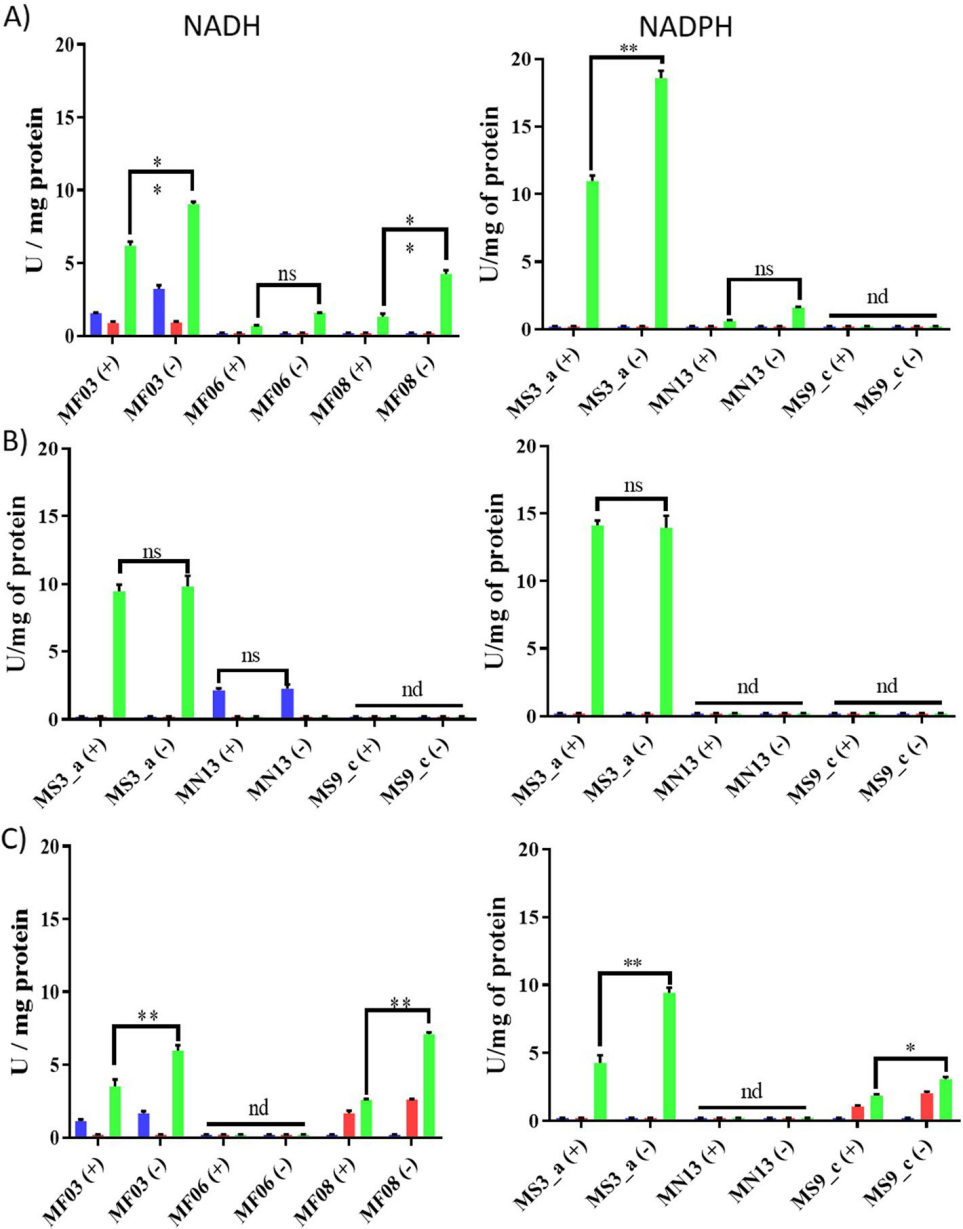
Metal(loid) reduction by *Exiguobacterium* strains under aerobic and anaerobic conditions. Reduction assays of Ag (**a**), Au (**b**) and Te (**c**) were carried out as described in Methods. Blue, red and green bars represent pH 7.0, 8.0 or 9.0, respectively. (+), aerobic test, (−) anaerobic test. Bars represent the average of 3 independent trials. **, *p* < 0.01; *, *p* < 0.05; nd, not determined; ns, not significant

Cell-free extracts were tested metal(loid) for reduction of Ag (I) (Fig. 1a), Au (III) (Fig. 1b), and Te (IV) (Fig. 1c). Extracts prepared from cultures in different growth phases were evaluated at 37 °C both in aerobic and anaerobic conditions at pH 7.0, 8.0, and 9.0, and in the presence of NADH or NADPH as electron donor. Hypothesizing that bioreduction is an enzymatic process in nature, denatured negative controls were conducted in which crude extracts were treated with 1% SDS or heated at 90 °C for 10 min prior to the assay (not shown). Metal(loid)-reducing activity was found to be higher in extracts from cells grown to mid exponential phase (OD_600_ 0.6) (not shown). While *E. acetylicum* MF03 silver-reducing activity was higher at pH 9.0, in anaerobiosis and NADPH as cofactor, *E. aurantiacum* MF06 showed higher Ag^+^-reducing activity at pH 9.0, irrespective of the electron donor or the presence of oxygen. In turn, *E. profundum* MF08 extracts showed higher activity at pH 9.0, with NADH under anaerobic conditions (Fig. 1a).

Regarding Au (III), there were no significant differences in reducing activity regarding the presence or absence of oxygen. Particularly, *E. acetylicum* MF03 displayed maximal gold-reducing activity at pH 9.0 and NADPH, whereas *E. aurantiacum* MF06 shower higher activity at pH 7.0 using NADH as the pyridine cofactor. *E. profundum* MF08 did not show Au (III)-reducing activity under the tested conditions (Fig. 1b).

Te (IV) was efficiently reduced by crude extracts of *E. acetylicum* MF03 (pH 9.0, NADPH, no oxygen) and *E. profundum* MF08 (pH 9.0, NADH, no oxygen, Fig. 1c). *E. aurantiacum* MF06 did not show tellurite reduction activity, irrespective of the tested condition.

**Fig. 2:**
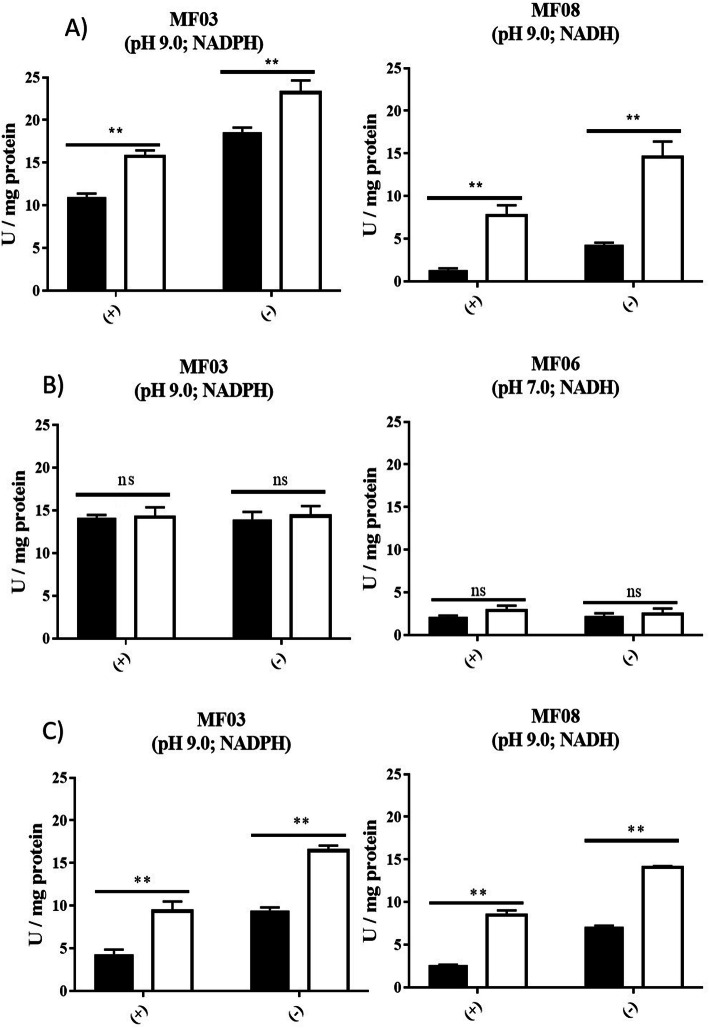
Assessing metal(loid)-reducing activity of crude extracts from *E. acetylicum* MF03, *E. aurantiacum* MF06 and *E. profundum* MF08. Reduction of Ag (**a**), Au (**b**) and Te (**c**) was carried out as described in Methods. Black and white bars represent no treatment or toxicant exposure, respectively. +, aerobic tests; −, anaerobic tests. Bars represent the average of 3 independent trials. **, p < 0.01; ns, not significant

Thus, *E. acetylicum* MF03 and *E. profundum* MF08 were used for silver reduction (Fig. 2a), *E. acetylicum* MF03 and *E. aurantiacum* MF06 for Au (III) reduction (Fig. 2b) and tellurite reduction was assessed using *E. acetylicum* MF03 and *E. profundum* MF08 (Fig. 2c).

To induce a cellular response by bacteria, a pretreatment with 1/8 of the MIC was performed for each toxicant; the aim was to favor the expression of genes encoding proteins involved in resistance to these elements, particularly reducing enzymes. At this concentration, a slight decrease of *Exiguobacterium* growth was observed regarding untreated control (data not shown). Extracts from *E. acetylicum* MF03 and *E. profundum* MF08 exposed to sublethal doses of silver salts showed increased Ag^+^-reducing activity regarding those grown in LB medium alone (Fig. 2a). On the other hand, no significant differences were observed in Au (III)-reducing activity by *E. acetylicum* MF03 or *E. aurantiacum* extracts under all conditions tested (Fig. 2b). Finally, and like Ag (I), Te (IV) reduction by extracts of *E. acetylicum* MF03 and *E. profundum* MF08 was more efficient than that observed in extracts prepared from cells not exposed to tellurite (Fig. 2c). With these results we were able to determine the optimal pH conditions and cofactor preference to be used later for NS synthesis.

### Generation and characterization of nanostructures

**Fig. 3:**
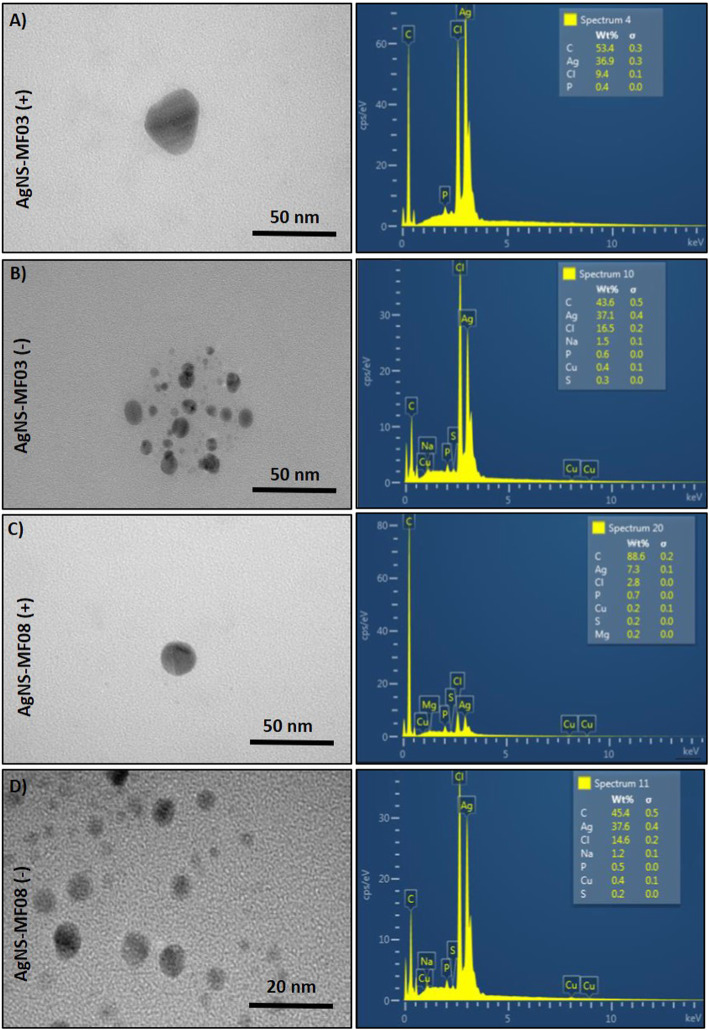
Characterization of silver nanostructures. Electronic micrographs (left) and EDS analysis (right) of in vitro generated AgNS, under anaerobic (**a**) and anaerobic conditions (**b**) by crude extracts of *E. acetylicum* MF03. **c** and **d**, AgNS generated aerobically and anaerobically, respectively, by *E. profundum* MF08

**Fig. 4:**
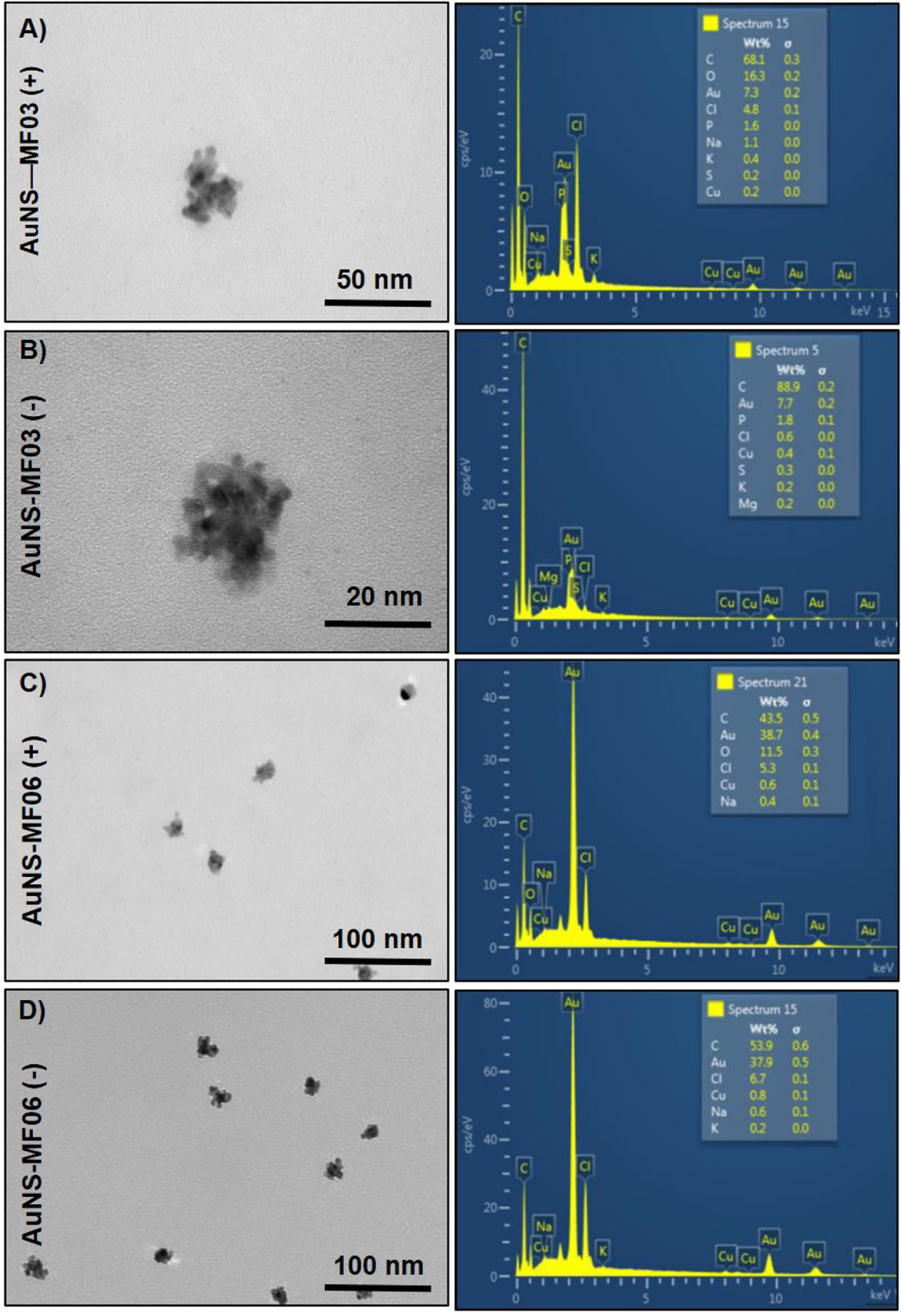
Characterization of gold nanostructures. Electron micrographs (left) and EDS analysis (right) of in vitro generated Au-NS, under aerobic (**a**) and anaerobic (**b**) conditions by crude extracts of *E. acetylicum* MF03; AuNS generated under aerobic (**c**) and anaerobic (**d**) conditions by *E. aurantiacum* MF06

**Fig. 5:**
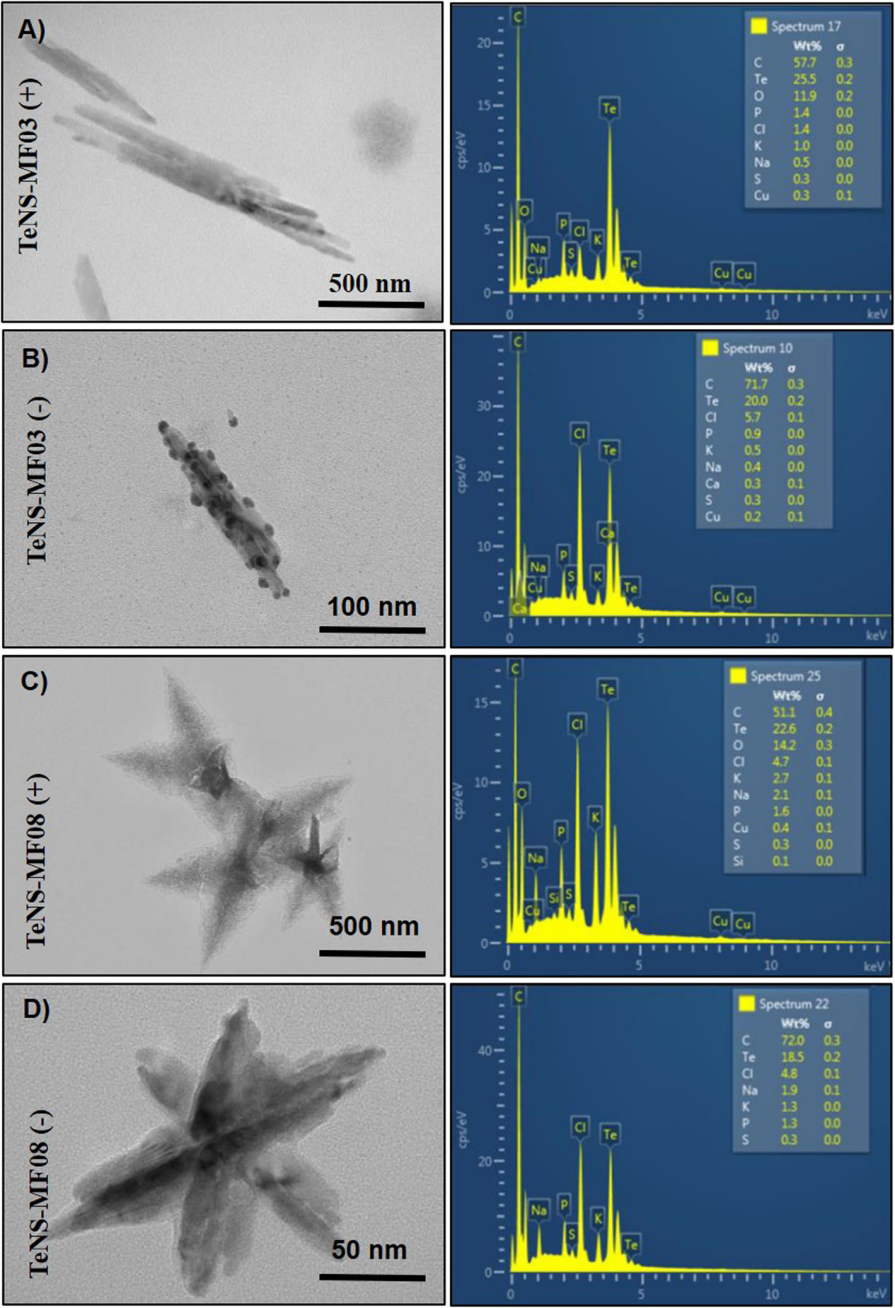
Characterization of tellurium nanostructures. Electronic micrographs (left) and EDS analysis (right) of NS from Te generated in vitro. TeNS generated aerobically (**a**) and anaerobically (**b**) by crude extracts of *E. acetylicum* MF03 and TeNS generated aerobically (**c**) and anaerobically (**d**) by *E. profundum* MF08

As above mentioned, conditions were set in reduction trials. Then, the ability of crude extracts from *Exiguobacterium* strains to generate NS -in the presence or absence of oxygen- was assessed. AgNS synthesis was conducted using crude extracts from *E. acetylicum* MF03 (pH 9.0, NADPH) or *E. profundum* MF08 (pH 9.0, NADH). In turn, while AuNS were generated using crude extracts of *E. acetylicum* MF03 (pH 9.0, NADPH) and *E. aurantiacum* MF06 (pH 7.0, NADH), TeNS formation was assessed using crude extracts of *E. acetylicum* MF03 (pH 9.0, NADPH) and *E. profundum* MF08 (pH 9.0, NADH). AgNS (Fig. 3), AuNS (Fig. 4) and TeNS (Fig. 5) relative size and morphology were characterized by transmission electron microscopy (TEM) and their chemical composition was assessed by energy dispersion spectroscopy (EDS).

Aerobically synthesized AgNS of ~ 40 nm by *E. acetylicum* MF03 showed triangular morphology (Fig. 3a), whereas those synthesized anaerobically were ~ 20 nm and were rather circular (Fig. 3b). These NS were composed mainly of carbon and silver both in aerobic (36.9% Ag) and anaerobic (37.1% Ag) conditions. On the other hand, AgNS generated by *E. profundum* MF08 in aerobic or anaerobic conditions exhibited circular morphology. While their size in aerobiosis was ~ 25 nm and were composed mainly of carbon (88%) and 7% silver (Fig. 3c), in anaerobic conditions they were ~ 15 nm with 45% carbon and 37.6% silver (Fig. 3d).

AuNS generated by *E. acetylicum* MF03 under aerobic (Fig. 4a) and anaerobic conditions (Fig. 4b) were ~ 25 nm and showed irregular morphology. They were composed mainly of carbon and gold (7.3 and 7.7%, respectively). On the other hand, AuNS generated by *E. aurantiacum* MF06, both in aerobiosis and anaerobiosis (Fig. 4), also exhibited irregular morphology of similar size (~ 25 nm), whose composition was mainly carbon with a gold content of 38.7% (aerobic, Fig. 4c) and 37.9% (anaerobic, Fig. 4d).

Finally, TeNS generated by *E. acetylicum* MF03 under aerobic and anaerobic conditions showed an elongated morphology of similar size, particularly in anaerobic conditions where they exhibited further electrodense zones. Their chemical composition was mainly carbon and tellurium (25.5%) under aerobic conditions. TeNS generated by *E. profundum* MF08 both in aerobiosis (Fig. 5c) and anaerobiosis (Fig. 5d) resulted in the formation of starred structures. However, their size in aerobiosis exceeded the nanoscale, being 10-fold bigger than their anaerobic counterparts (~ 100 nm).

## Discussion

Based on 16S rRNA phylogenetic studies of *Exiguobacteriu*m genus, it has shown that both *E. acetylicum* and *E. profundum* display three branch points from the common ancestor with *E. aurantiacum*. Additionally, *E. profundum* and *E. aurantiacum* show privileged homology since they are clustered in a joint node, despite showing higher speciation [3]. At the genome level, the draft sequence of *E. aurantiacum*, *E. profundum* [31] and the sequence/assembly of *E. acetylicum* are available in the databases.

The distinctive feature belonging to this genus is their ability to grow under extreme environmental conditions, including a wide temperature range (− 12–55 °C) besides nutrient limitation situations [32, 33]. The robustness and tolerance against harsh conditions showed by *Exiguobacterium* members turn them in suitable candidates for industrial applications, useful in bioremediation and bioabsorption processes of metals and metalloids [34–37].

*Exiguobacterium* strains used in this research were previously isolated from different regions in Chile that display a combination of extreme environments such as high salinity, desiccation, high and low temperatures, and volcanic interventions [38]. Most of these factors are responsible for generating oxidative stress to microorganisms, a situation that also occurs often upon bacterial exposure to metal(loid)s. This is corroborated, because in Chile it has been identified and characterized strains of *Exiguobacterium* obtained from the Salar del Huasco which have arsenic resistance [1, 2].

In this work, resistance for the three elements was higher in the absence of oxygen, a result that was expected because of the absence of ROS, which otherwise would generate oxidative stress [14, 29].

To determine optimal parameters, reduction assays of TeO_3_^2−^, AuCl^4−^ and Ag^+^ by crude extracts were carried out at pH values 7.0–9.0, in the presence of NADH or NADPH as the electron donor. Particularly, this study worked in the optimal temperature conditions (37 °C), however, it would be interesting to study the minimum and maximum ranges that the system supports, since *Exiguobacterium* is a polyextremophilic microorganism. Crude extract-mediated reducing activity was higher at pH 9.0 for most tested metal(loid)s, irrespective of the presence of oxygen (Fig. 1). This could reflect the fact that most proteins displaying metal(loid)-reducing activity contain catalytic sites including vicinal cysteine residues that play an important role in the reduction process [39]. Therefore, at basic pH, deprotonation of thiol groups from these cysteines would occur, giving rise to the highly reactive thiolate anion (−S^−^) [40]. Another possible explanation is that enzymes exhibiting metal(loid)-reducing activity are tolerant to alkali, as has been described for most enzymes of biotechnological interest isolated from *Exiguobacterium* strains [3]. In turn, *E. aurantiacum* MF06 crude extracts showed Au (III)-reducing activity at pH 7.0 (Fig. 1b), a situation that may occur because pH can influence metal(loid) speciation. This could result in the formation of complexes and/or deprotonation or protonation of functional groups in amino acids that participate in enzyme-substrate stabilization [41] (Panda and Deepa, 2011). The preference for NADH or NADPH as electron donor could reflect its stabilization at the enzyme’s active site [42].

For Ag^+^- and TeO_3_^2−^-reducing activities, these were higher under anaerobic conditions, probably due to the limitation of ROS formation in this condition [14, 29]. To date, gold toxicity has not been associated to oxidative stress; the lack of significant differences in AuCl_4_^−^ reduction by crude extracts from aerobically- or anaerobically grown cells supports this observation.

Assays that were carried out with crude extracts from cells that were previously exposed with sublethal doses of toxicants showed -in general- higher reducing activities than those coming from untreated cells both under aerobic and anaerobic conditions with the exception of Au (III) reduction. Since bacterial Ag and Te resistance is associated to enzymatic reduction [14], the observed results could reflect the expression of genes related to bacterial Ag (I) and/or Te (IV) resistance.

In addition, crude extracts of this genus have been previously used for nanoparticle synthesis. For instance, *E. mexicanum* extracts were able to synthesize silver nanoparticles of 5–40 nm, a process in which extracellular polymeric substances played a critical role both in silver reduction and nanoparticle stabilization [12]. Because of this, we used *Exiguobacterium* strains as an ecofriendly way to get NS.

NS synthesis by bacterial crude extracts or purified enzymes has not been widely reported. Indeed, most synthetic procedures are chemical in nature, in which mechanisms of NS formation involve two stages: nucleation and growth, processes that are affected by several factors including thermal energy, metal concentration and reaction rate, among others [43].

AgNS synthesized in aerobic conditions using crude extracts of *E. acetylicum* MF03 and *E. profundum* MF08 exhibited larger sizes than their anaerobic counterparts. However, the highest silver-reducing activity was observed precisely in the absence of oxygen. Given that, NS size could be affected by the activity of the enzyme, the following tests considered protein concentration as a critical parameter. Indeed, it was previously observed that during enzymatic synthesis of tellurium-containing NS, particle size was inversely related to enzyme concentration [26]. In addition, NS yield was higher under anaerobic conditions. These results could be explained by a higher metal(loid)-reducing as result of the absence of oxygen that could prevent electron leakage [44].

On the other hand, AuNS generated by *E. acetylicum* MF03 and *E. aurantiacum* MF06 did not show significant fluctuations in size or yield both in aerobiosis and anaerobiosis. However, what is relevant about these results is that when working with two different species of *Exiguobacterium* it is possible to obtain AuNS with different gold content, which from a biotechnological point of view is attractive, for example in the field of medicine.

Finally, aerobically- and anaerobically generated TeNS by *E. acetylicum* MF03 displayed similar sizes along with elongated morphologies; the exception was anaerobically-synthesized TeNS, which showed dense electron spots. This kind of elongated morphology of tellurium-containing NS was previously reported for *Rhodobacter capsulatus* [45]. In turn, TeNS generated by *E. profundum* MF08 (Fig. 5c-d) were much larger in aerobic conditions, which does not correlate with the reducing activity of crude extracts. Similarly, to what was proposed for AgNS, TeNs could be adopting different nucleation/growth processes that could explain this observation [43].

In general, the composition analysis of in vitro synthesized NS included the metal(loid) itself along with other, apparently unrelated elements. These include mainly carbon, oxygen and sulfur, probably indicating the organic origin of NS formation. Consistent with this, previous studies have shown that AuNS can be found in association with the enzyme glutathione reductase [46].

Biological processes for NS synthesis remain a challenge, not solely as a synthetic platform but in green purification techniques for subsequent characterizations. More efforts should be made to expand the characterization techniques applicable to these methods such as XRD, DLS with potential Z, FTIR, among others. When irregular NS with variable size and undefined organic layers are obtained, the results of these analyzes generate errors so they might be not reliable. In our case, XRD analyzes were not possible to perform because the surface of the nanostructures was not clean enough due to the biological processes that were used for the synthesis, this can be seen in the EDS analyzes in which many elements, of organic origin, they are identified, so the background is abundant. However, during TEM observation and navigation, SAED and FFT (Fourier transform) electron diffraction were explored, revealing the polycrystalline character of the samples without identifying preferential growth axes or phenomena of crystallographic and significant interest to report.

All these results allow us to demonstrate the great applicability of the *Exiguobacterium* genus in processes of resistance, reduction and generation of NS of metals and metalloids, which could be applied to help in developing effective co-cultures to improve the metal(loid) polluted sites like those described in Batool et al. [47]. Moreover, developing new bacterial-assisted techniques for reduced metal(loid) uptake of vegetables in the metal(loid)-contaminated soils [48].

## Conclusion

The procedures of this study, related to NS synthesis of Ag, Au or Te obtained with *Exiguobacterium* strains under aerobic or anaerobic conditions opens the possibility of future controlled biological approaches which represent an interesting green methodology in the field of nanotechnology.

## Methods

### Bacterial strains and culture conditions

Bacterial strains *Exiguobacterium acetylicum* MF03, *E. aurantiacum* MF06 and *E. profundum* MF08 used in this study were previously characterized [38]. Cells were grown in the presence or absence of oxygen at 37 °C in Luria-Bertani culture medium (LB) with constant shaking (150 rpm) from 1% inoculum of a preculture grown overnight (approx 10^9^ CFU/ml). Anaerobic assays were conducted inside a Coy chamber (Coy Laboratory Products, Inc.®), which provides a strict anaerobic environment (100% N_2_). Solutions, buffers and culture media (solid or liquid) were equilibrated before their use in the anaerobic atmosphere by introducing them to the chamber for at least 12 h for liquid medium and solutions and 3 h for solid media.

In liquid grown condition times required to achieve the optical density of 0.6 (mid-exponential phase), at 600 nm of wavelength (OD_600_), under aerobic conditions are 3, 6, and 4 h for *E. acetylicum* MF03, *E. aurantiacum* MF06, and *E. profundum* MF08, respectively. On the other hand, under anaerobic conditions all strains required an extra hour to reach the mid-exponential phase.

### Determination of the minimal inhibitory concentration (MIC)

Using sterile stock solutions of 40 mM K_2_TeO_3_ [Te (IV)], 50 mM AgNO_3_ [Ag (I)] and 10 mM HAuCl_4_ [Au (III)], serial dilutions were made in 1 ml of LB medium using 48-well culture plates. Then, 10 μL of cells previously grown in LB to OD_600_ 0.6 were added to each well and incubated with constant shaking (150 rpm) at 37 °C. Minimal inhibitory concentrations (MICs) were defined as the lowest concentration that inhibits the visible growth of a microorganism, and were determined as the average of six independent trials (*n* = 6) by monitoring turbidity at 600 nm after 24 h as described by Fuentes et al [49].

### Crude extract preparation

Cultures were grown to OD_600_ 0.6 at 37 °C in the absence or presence of a sublethal dose of the toxicant to be tested (1/8 MIC) and centrifuged at 9000 x *g* for 5 min at 4 °C. After discarding supernatants, sediments were suspended in 50 mM Tris-HCl buffer pH 7.0, 8.0 or 9.0, containing 0.1 mM PMSF. Cell suspensions were sonicated on ice with 4 pulses of 20 s each at 60% amplitude. The cell debris was discarded by centrifuging at 14,000 x *g* for 10 min at 4 °C and supernatants, containing soluble proteins, were collected and considered the crude extracts.

### Determination of protein concentration

Protein concentration was quantified accordingly to Bradford protocol [50] using bovine serum albumin (BSA) as standard; the absorbance at 595 nm was determined using a Tecan INFINITE M200 Pro multiplate reader.

### Determination of enzyme activity

Metal(loid)-reducing activity present in crude extracts was determined at 37 °C in a final volume of 200 μl which contained 20 μL of the corresponding extract in 50 mM Tris-HCl buffer pH 7.0, 8.0 or 9.0, 1 mM NAD(P) H, 1 mM β-mercaptoethanol and the toxicant to be evaluated (1 mM K_2_TeO_3_, 0.2 mM AgNO_3_ or 1 mM HAuCl_4_). Metal(loid) reduction was monitored at the highest absorption wavelength of the metal(loid) in the zero-state (500, 424 and 540 nm for Te, Ag and Au, respectively) using a Tecan multiplate reader INFINITE M200 Pro equipment. All experiments were performed in triplicate and with their respective controls. Controls excluding extracts were run in each case to rule out abiotic reduction.

An enzyme unit (U) was defined as the amount of enzyme required to increase the absorbance by 0.001 units/min at the respective wavelength. Similar reaction mixes -but containing 200 μg/ml protein- were used for NS biosynthesis. Optimal pH and electron donor concentration were determined, and reactions lasted 18 h.

### In vitro synthesis and characterization of NS

Crude extracts from each strain (200 μg/mL protein) were used to produce nanostructures by incubation with 1 mM mM K_2_TeO_3_, 0.2 mM AgNO_3_ or 1 mM HAuCl_4_ for 16 h at the optimal conditions of temperature, pH, cofactor and growth conditions (as determined from reduction assays) in a final volume of 1 mL. Controls to rule out abiotic synthesis were run in each case.

The morphology of the synthesized nanostructures was analyzed by Transmission Electron Microscopy (TEM) using a Hitachi Transmission Electron Microscope HT7700 equipped with a thermionic Lanthanum Hexaboride (LaB6) filament under 120 kV of acceleration voltage. Each sample was prepared by placing a drop (20 μl) of nanostructure suspension on a 250-mesh copper grid previously covered with a carbon film. The NS chemical composition as determined by Energy dispersive X-ray spectroscopy (EDS) analysis; briefly, nanostructure suspensions were centrifuged at 13,000 x *g* for 60 min and after drying the sediment for 1 h at 40 °C, samples (approximately 20 μl), as well as their respective controls, were supported on glass slides and stained using the Gram-Hucker kit. Then they were fixed on conductive adhesive over pin stub mount and coated with a gold film to protect the surface from damage and calcinations and to minimize charge related artifacts. Analyses by Scanning Electron Microscopy (SEM) were carried out using a Zeiss EVO MA-10 microscope with a tungsten filament gun and by EDX spectra. Data were collected using an Oxford Instruments X-act system (connected to a microscope equipped with a Penta FET Precision detector). Samples were imaged at an accelerating voltage of 20 kV and 8 mm of working distance. All studies of electron microscopy were conducted at the Center for the Development of Nanoscience and Nanotechnology–CEDENNA, USACH.

### Data analysis

Statistical analysis and graphs were carried out using GraphPad Prism 6.0 (GraphPad Software, Inc.). The confidence interval in the analysis of variance (ANOVA) was set at *p* < 0.05. The statistical significance was indicated as follows: ∗, p < 0.05, ∗∗, *p* < 0.01, ∗∗∗, *p* < 0.001 and ∗∗∗∗, *p* < 0.0001; ns, not significant.

Raw data from analyzes are in the supplementary information file named “Availability of Data”.

## Supplementary information

**Additional file 1.Figure 1S.** Growth inhibition zones of the strains belonging to the *Exiguobacterium* genus exposed to Ag(I), Au(III) and Te(IV). Growth inhibition areas for *E. acetylicum* MF03 [A], *E. aurantiacum* MF06 [B] and *E. profundum* MF08 [C] were determined under aerobic (blue) and anaerobic (red) growth conditions. Bars indicate an average of 6 independent tests ± standard deviation. ****, Indicates significant statistical difference (*p* <0.0001) and ns, not significant.

**Additional file 2.** Availability of Data.

## Data Availability

All data generated or analyzed during this study are included in this published article and its supplementary information files.

## Abbreviations

AgNS: Silver nanostructure
AuNS: Gold nanostructure
EDX or EDS: X-ray energy dispersion spectroscopy
MIC: Minimum inhibitory concentration
NADH: Nicotinamide adenine dinucleotide
NADPH: Nicotinamide adenine dinucleotide phosphate
NS: Nanostructure
OD: Optical density
PMSF: Phenylmethylsulfonyl fluoride
SEM: Scanning electronic microscopy
TEM: Transmission electronic microscopy
TeNS: Tellurium nanostructure
