# Supplementary material for: In vitro biosynthesis of Ag, Au and Te-containing nanostructures by Exiguobacterium cell-free extracts
Source: BMC Biotechnol. 2020 May 29;20:29. doi: 10.1186/s12896-020-00625-y (PMC7260758; doi:10.1186/s12896-020-00625-y)
Supplement: Supplementary file 1 — Additional file 1.Figure 1S. Growth inhibition zones of the strains belonging to the Exiguobacterium genus exposed to Ag(I), Au(III) and Te(IV). Growth inhibition areas for E. acetylicum MF03 [A], E. aurantiacum MF06 [B] and E. profundum MF08 [C] were determined under aerobic (blue) and anaerobic (red) growth conditions. Bars indicate an average of 6 independent tests ± standard deviation. ****, Indicates significant statistical difference (p <0.0001) and ns, not significant. [file 12896_2020_625_MOESM1_ESM.docx]

Supplementary Material

***In vitro* biosynthesis of Ag, Au and Te-containing nanostructures by *Exiguobacterium* cell-free extracts**

Orizola J., Ríos-Silva M., Muñoz-Villagrán C., Vargas E., Vásquez C. and Arenas F.

**Correspondence to:** Felipe A. Arenas

E-mails: felipe.arenass@usach.cl


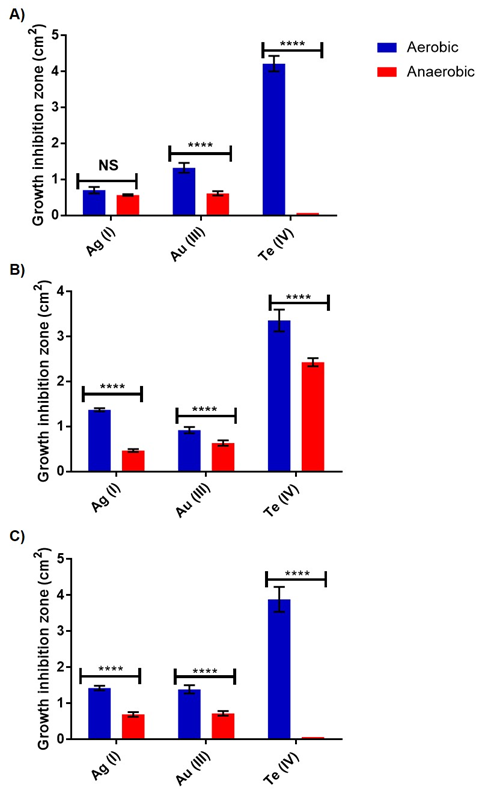


**Figure 1S. Growth inhibition zones of the strains belonging to the *Exiguobacterium* genus exposed to Ag(I), Au(III) and Te(IV).** Growth inhibition areas for *E. acetylicum* MF03 [A], *E. aurantiacum* MF06 [B] and *E. profundum* MF08 [C] were determined under aerobic (blue) and anaerobic (red) growth conditions. Bars indicate an average of 6 independent tests ± standard deviation. ****, Indicates significant statistical difference (p <0.0001) and ns, not significant.
